# Impact of preconception enrollment on birth enrollment and timing of exposure assessment in the initial vanguard cohort of the U.S. National Children’s Study

**DOI:** 10.1186/s12874-015-0067-1

**Published:** 2015-09-24

**Authors:** Joseph B. Stanford, Ruth Brenner, David Fetterer, Leslie Palmer, Kenneth C. Schoendorf, for the U.S. National Children’s Study

**Affiliations:** Department of Family and Preventive Medicine, University of Utah, 375 Chipeta Way, Salt Lake City, UT 84108 USA; Department of Pediatrics, University of Utah, Salt Lake City, UT USA; Eunice Kennedy Shriver National Institute of Child Health and Human Development, Bethesda, MD USA; ARCBridge Consulting and Training, Herndon, VA USA; Greenspring Pediatric Associates, Sinai Hospital of Baltimore, Baltimore, MD USA

**Keywords:** Birth cohort, Children, Epidemiology, National children’s study, Pregnancy, Prospective studies, Intention

## Abstract

**Background:**

The initial vanguard cohort of the U.S. National Children’s Study was a pregnancy and birth cohort study that sought to enroll some women prior to pregnancy, and to assess exposures early in pregnancy.

**Methods:**

During the recruitment phase (2009–2010), geographically based sampling was used to recruit women early in pregnancy and women not currently pregnant, not using contraception and heterosexually active. We assessed the following outcomes for women enrolled preconception and early in pregnancy: yield of births; demographic characteristics of births for different enrollment groups; time to pregnancy for preconception women; and the timing of study visits for exposure assessment.

**Results:**

1399 women were recruited into the initial vanguard cohort: 429 preconception (198 trying for pregnancy, and 231 not trying) and 970 already pregnant. There were 1135 pregnancies (81 % of women) and 922 newborns enrolled (81 % of pregnancies) through September 2012. Preconception women represented 30.6 % of women enrolled, and contributed 14.5 % of births. Among women who gave birth, and who had enrolled preconception trying for pregnancy, 67.3 % were white non-Hispanic, compared to 50.0 % of preconception women not trying for pregnancy, and 61.5 % of pregnant women. Women enrolled preconception who were trying for pregnancy had higher cumulative probability of pregnancy at one year compared to women not trying (adjusted 86 % versus 56 %). Of 165 women enrolled preconception who became pregnant, 19 % had a study visit within 30 days of conception. By 10.5 weeks after conception, 75 % of women enrolled preconception had completed a pregnancy study visit; for women enrolled pregnant, the 75 % threshold was reached at 28.4 weeks.

**Conclusions:**

There were demographic differences in births from women enrolled preconception trying for pregnancy, preconception not trying for pregnancy, or during pregnancy. Time to pregnancy was shorter for women actively trying for pregnancy. Most women enrolled preconception did not have exposure assessment within 30 days of conception, but they did have exposure assessment much earlier during pregnancy than women who enrolled during pregnancy.

## Background

The United States National Children’s Study (NCS) was planned as a longitudinal study designed to examine effects of environmental exposures on children’s health [1]. As defined by the NCS, “environment” is conceived broadly to include chemical, physical, psychosocial, and biological exposures as well as gene environment interactions and epigenetic influences. The NCS used a probability sampling scheme designed to be representative of the United States population [2, 3]. The study has now been discontinued, but data from its initial phases are available for analysis [4].

The NCS began with a pilot or vanguard phase, starting with seven Initial Vanguard Centers, with later expansion to add 33 more Vanguard Centers. The purpose of the initial vanguard cohort of the NCS has been to develop and pilot test methods to be used in the NCS, with emphasis on feasibility, acceptability, and costs of recruitment, logistics, and study visit assessments [1, 5, 6].

In the initial planning stages of the NCS, some of the proposed innovations of the NCS relative to other birth cohorts included 1) rigorous population-representative sampling, 2) very detailed assessments of environmental exposure, and 3) preconception and early pregnancy enrollment for more timely and accurate exposure assessment around conception (fertilization) and the early critical windows of embryonic and fetal development [7]. All of these features were emphasized in the initial vanguard cohort of the NCS; the third point is the main focus of this paper.

To meet the scientific objective of prospective assessment of periconception, embryonic, and early fetal exposure, the initial vanguard cohort of the NCS was designed to enroll women as early in pregnancy as possible, and also to enroll some women who were not currently pregnant, but heterosexually active and at high probability of becoming pregnant in the near future [8]. A number of questions arose about the impact of preconception recruitment, including the yield of births, and the potential for selection bias from recruiting women who self-identify as trying for pregnancy, and the potential for timely completion of exposure assessments, particularly preceding and/or following the estimated time of conception [9, 10].

In this paper, we use data assembled by the seven Initial Vanguard Centers (recruited in 2009–2010) to assess the impact of preconception and early pregnancy recruitment in the initial vanguard cohort of the National Children’s Study on the following study outcomes:

1. Yield of births in the study from women enrolled preconception and during early pregnancy, assessing efficiency for births enrolled.
2. Demographic characteristics of births from women enrolled preconception and during early pregnancy, assessing representativeness of the different types of enrollment. This included two subtypes of women at high probability of pregnancy: those who self-identified as “trying” to become pregnant and those who were not “trying” for pregnancy but still at behavioral risk of pregnancy.
3. Time to pregnancy for women identified on initial population screening as having high probability of pregnancy in the near future (including both subtypes), assessing time required for preconception recruitment.
4. Time from first study visit to conception for women enrolled preconception, assessing timeliness of preconception exposure assessment.
5. Time from conception to the next study visit (which could be the first study visit) for all women in the study, assessing timeliness of early pregnancy exposure assessment.

## Methods

### Sampling

Details of the initial sampling strategy for the NCS have been previously reported [3, 11]. Briefly, 105 primary sampling units (PSUs) were identified involving 110 counties. Subsequently, 7 Initial Vanguard Centers were chosen to implement the initial vanguard cohort of the NCS in 7 PSUs: the Mount Sinai School of Medicine (Queens County, NY), the Children’s Hospital of Philadelphia (Montgomery County, PA), the University of Wisconsin (Waukesha County, WI), South Dakota State University (Brookings County SD, Yellow Medicine County MN, Pipestone County MN and Lincoln County MN), the University of California at Irvine (Orange County, CA), University of Utah (Salt Lake County, UT), and the University of North Carolina at Chapel Hill (Duplin County, NC). Within each PSU, smaller geographic areas (termed segments) were selected as the secondary sampling units (SSU), using a stratified random sampling scheme.

### Timeline

**Fig. 1:**
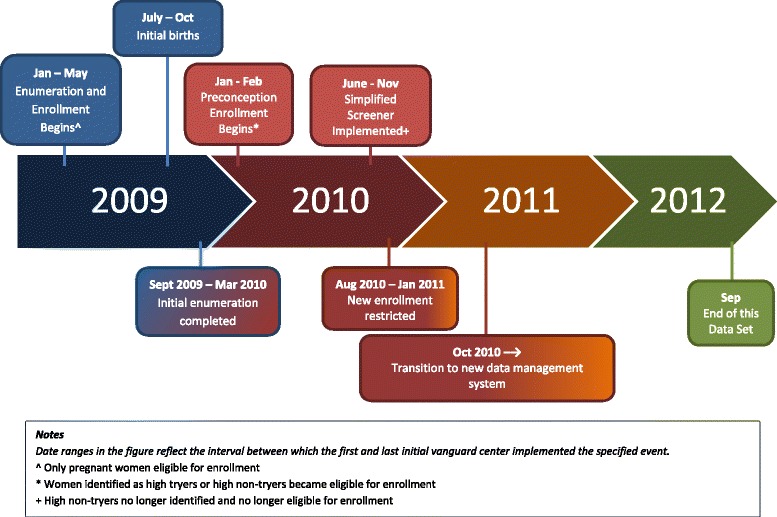
Key events and changes in eligibility during the enrollment period for the initial vanguard centers of the National Children’s Study

A timeline of key events related to study enrollment, changes in eligibility for enrollment, and other events related to this analysis is given in Fig. 1. Each of the events noted there is described in more detail below.

### Enumeration, eligibility screening and enrollment

Enumeration, screening for eligibility and enrollment began in January 2009 for two Initial Vanguard Centers, and was implemented in all seven Initial Vanguard Centers by May 2009, as has been described in detail previously [11]. The primary means of identifying and recruiting eligible women was based on contact at residences within each SSU. Data collectors went door to door to each household and enumerated all persons living in the household, based on one adult informant.

Women of reproductive age living in the household were screened for eligibility via a “pregnancy screener.” The pregnancy screener included a computer-assisted self interview with audio that asked questions addressing pregnancy, sexual activity, use of birth control, and medical history of infertility or sterility. Initially, only pregnant women who were within the first 20 weeks of pregnancy and residing in a SSU were eligible for inclusion in the study; after approximately 6 months from when each individual SSU began enrollment any pregnant woman residing in that SSU became eligible. Based on local IRB requirements, some Initial Vanguard Centers enrolled pregnant women under age 18, and some did not.

The initial enumeration of all geographically eligible households lasted approximately 6–8 months, with some variability among the Initial Vanguard Centers. Both concurrently and subsequently, other awareness and recruitment activities were implemented to identify new households and new women moving into established households, as well as strategies to promote the self-identification of newly eligible women to NCS staff, including promotion of the study at clinics, community outreach activities and referral from study participants. The precise mix of activities varied according to local circumstances of the Initial Vanguard Centers.

Women who were not pregnant, and who were not sterile were asked for permission to be followed with periodic telephone calls. These women were classified as having high, moderate, or low probability of pregnancy, with the frequency of the follow-up calls varying according to their expected probability of becoming pregnant, as described in more detail elsewhere [11]. Women were classified as “high probability of pregnancy” if they were age 18–44, not currently pregnant, and trying to get pregnant for less than 5 months, i.e., “high tryers.” Women who were age 18–34 years, heterosexually active (within the past 3 months), and not using any birth control method (other than withdrawal or natural family planning) were classified as “high non-tryers”. Both of these groups were contacted one, two, and four months after being so identified. Starting in June 2010 (up to November 2010 for some centers), the follow-up telephone pregnancy screener was simplified to remove any questions about birth control use or sexual activity, and women were eligible for preconception enrollment only if they indicated they were currently trying to become pregnant for less than 5 months (i.e., high non-tryers were no longer identified or offered preconception enrollment). Women classified as having a moderate probability of pregnancy were called every 3 months, and women classified as having a low probability of pregnancy were contacted in 6 months. Women who were identified at a follow-up telephone call as eligible (pregnant or newly at high probability of pregnancy) were recruited for the study.

Informed consent was obtained in person from preconception and pregnant women for their participation in the study. During the third trimester, or at the time of birth, an additional informed consent was obtained to allow the infant to continue in the study. The initial vanguard study protocol and all subsequent amendments were approved by the *Eunice Kennedy Shriver* National Institute of Child Health and Human Development (NICHD) Institutional Review Board (protocol # 09-CH-N083), and local Institutional Review Boards at each of the participating Initial Vanguard Centers: the Icahn School of Medicine at Mount Sinai Institutional Review Board; the Children’s Hospital of Philadelphia Research Institute Committee for the Protection of Human Subjects (IRB); the University of Wisconsin-Madison Health Sciences Institutional Review Board; South Dakota State University Human Subjects Research Committee; Institutional Review Board A, Human Research Protection Program, Office of Research Administration, University of California, Irvine; the University of Utah Institutional Review Board; the Non-Biomedical Institutional Review Board at the University of North Carolina, Chapel Hill.

### Preconception enrollment

While enrollment of pregnant women began in January-May 2009, preconception enrollment was begun in January 2010. Women identified as high-tryers or high non-tryers were eligible for preconception enrollment and data collection. After 4 months in either high probability group without pregnancy, women were transitioned to a moderate probability group and were no longer eligible for preconception enrollment (but still eligible for pregnancy enrollment).

### Restriction of recruitment and transition to retention

Recruitment and enrollment continued through September 2010. Thereafter, the protocol was streamlined considerably; during this time new recruitment was de-emphasized and ultimately phased out for the Initial Vanguard Centers. Although some women ultimately enrolled more than one child in the NCS, only first pregnancies of the women in the initial vanguard cohort are included in this analysis. The closing date for the data set used for this analysis is September 2012. We are aware of 9 additional births to this cohort after that date that are not included in this data set.

### Identification of the estimated date of conception and time to pregnancy

The estimated date of delivery, or “due date,” was taken from ultrasound records. The study protocol specified that one ultrasound should be done in each trimester. Some of these ultrasounds were done by the study, and some were done as part of clinical care; in the latter case, clinical ultrasound reports were obtained and data were abstracted. The earliest ultrasound available was used for determining the estimated date of delivery. The estimated date of conception (fertilization) was calculated as the date exactly 38 weeks prior to the due date.

### Study home visits

Following informed consent, separate home visits were scheduled for the initial data collection. The schedule of home visits in the study included preconception visits, first trimester visits, and third trimester visits. The stage in her pregnancy when a woman consented (e.g., preconception, first trimester, or later) determined which of these would be her first study visit; she was then eligible for all subsequent visits on the study schedule. Each of these home visits lasted approximately 3 h to complete all interview, anthropometric, biological and environmental assessments. The study also included separate ultrasound visits, birth visits (in the hospital or at home), subsequent newborn visits, and telephone contacts about the newborn; but these visits are not considered in the scope of this paper.

Additional details of the design of the initial vanguard cohort of the NCS have been reviewed elsewhere [11, 12].

### Statistical analysis

We calculated descriptive statistics to describe the proportions of participants conceiving and giving birth, and to summarize their demographic characteristics. We examined the distributions of the following time intervals: 1) time from screening to pregnancy for women found to be at high probability of pregnancy at initial screening, 2) time from the preconception visit to conception for women enrolled preconception, and 3) time from conception to the first study visit for all women in the study. For time from screening and time from conception to the first study visit, we employed survival analysis to account for those exiting from follow up (due to moving, loss to follow up, pregnancy loss, or withdrawal). Also censored in the analysis of time to first home study visit are those still awaiting a visit at the point of transition to a new study data system after September 2010, since we did not have data on visits available from that system. For some analyses, we excluded a small number of participants who had date inconsistencies that could not be resolved with available data (for example, a pregnancy home visit that occurred more than a year after the date of conception).

## Results

### Yield and retention

From the beginning of enrollment (January 2009 through May 2009 for the different Initial Vanguard Centers) through September 2010, 30,062 women were screened, of whom 744 were pregnant at the time of screening, and 23,608 were eligible for telephone follow-up based on their geographic residence, age and reproductive potential. Among these women, 831 became pregnant (and eligible) during the follow-up recruitment period, and 861 became eligible because of transitioning to a high probability of conceiving. Ultimately, 1399 women were enrolled into the initial vanguard cohort, representing 61 % of the women found eligible for the study. Details of screening, eligibility, and enrollment of all women screened for the study have been reported previously [11]. Table 1 summarizes the proportion of preconception women and pregnant women enrolled in the study, and the resulting births. Preconception women represented 30.6 % of the women consented for the study, but contributed 15.4 % of the pregnancies and births in the study. Pregnancy loss was documented for 34 women (3.0 % of pregnancies overall); however, we believe that there were additional pregnancy losses that were lost to follow-up, for reasons described in the discussion. Among the 1399 women enrolled in the study, we had missing information for 0 (0 %) for date of birth, and 66 (4.7 %) for race or ethnicity.

**Table 1:** Enrollment, pregnancy, and births by eligibility status at initial consent

	High non-tryer	High tryer	Total preconception	Pregnant	Total
Number of women consented	231	198	429	970	1399
Proportion of women consented in study	16.5 %	14.2 %	30.6 %	69.3 %	100.0 %
Number of pregnancies	54	111	165	970	1135
Proportion of women conceiving	23.4 %	56.1 %	38.5 %	100.0 %	81.1 %
Proportion of pregnancies in study	4.8 %	9.8 %	14.5 %	85.5 %	100.0 %
Number of women giving birth	44	98	142	780	922
Proportion of pregnant women with known birth	81.5 %	88.3 %	86.1 %	80.4 %	81.2 %
Proportion of births in study	4.8 %	10.6 %	15.4 %	84.6 %	100.0 %

Eligibility status at consent often differed from eligibility status at original screening because of a delay in implementing preconception enrollment. Of the 970 enrolled pregnant, 228 were not pregnant at initial screening. See Fig. 1 and reference #11

Of all women who were pregnant and enrolled in the initial vanguard cohort, 81.2 % had a birth with an infant identified by the study. All but 21 (2.2 %) of the 922 births identified in the study follow-up had documented consent to continue in the study. The earliest study birth occurred in July 2009, and the latest study birth in this data set occurred in September 2012. Of the 922 births in the initial vanguard cohort, 11 % occurred in 2009, 64 % in 2010, 18 % in 2011, and 2 % in 2012, with 5 % having an unknown date of birth.

### Demographic characteristics by type of eligibility at consent

Table 2 depicts maternal age and race/ethnicity distributions among NCS births, in relation to the eligibility status at the time of consent. We note that women under 18 years of age were eligible for enrollment for the NCS only if they were already pregnant. However, because some cell counts are too small they are not displayed in accordance with NCS non-disclosure policy. High tryers were less likely to be Hispanic. There were no high tryers ages 18–19. Despite age eligibility restriction, there were some high non-tryers who were 35 or older (albeit too few to display). High non-tryers were much less likely to be white, non-Hispanic than either high tryers or pregnant women.

**Table 2:** Age and race/ethnicity of women giving birth in the NCS by eligibility status at the time of consent

		Status at consent		
		High non-tryer	High tryer	Pregnant
		%	%	%
Age, years	<18	0	0	1.4
	18–19	*	0	3.3
	20–34	90.9	83.7	76.5
	35+	*	16.3	18.7
	Mean (standard deviation)	27.7 (4.0)	30.7 (4.1)	29.2 (5.7)
Race/ethnicity	Asian, non-Hispanic	*	*	4.2
	Black, non-Hispanic	*	*	6.4
	Hispanic	22.7	10.2	20.0
	Other^	20.5	20.4	8.0
	White, non-Hispanic	50.0	67.3	61.5

*Cell counts are too small to display. (Despite age eligibility criteria for high non-tryers, the cell counts for age 35+ are not zero)

^includes women who report two or more races. For age categories, *P* < 0.05; For race and ethnicity, *P* < 0.01

### Time to pregnancy among preconception women

**Fig. 2:**
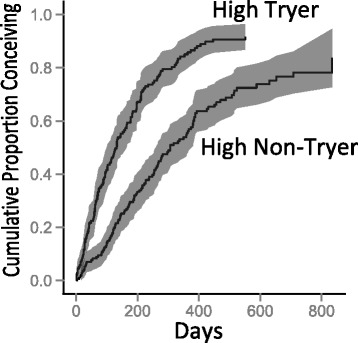
Time from screening to conception by pregnancy probability group (High Tryer versus High Non-Tryer) at initial screening, adjusted for loss to follow-up. Shading represents 95 % confidence interval

We assessed time to pregnancy for all the women identified as preconception eligible at screening. (Many of these women did not actually enroll preconception, but rather enrolled when they were already pregnant, because their screening happened prior to the implementation of the preconception part of the study in January 2010.) Of the 1399 women who ultimately enrolled, 403 women were identified *at the time of initial screening* as eligible for preconception enrollment (215 high non-tryers, and 188 tryers). Of these women, 16 high non-tryers and 23 high tryers subsequently had an estimated date of conception determined that was on or prior to the date of screening (i.e., they were actually pregnant at screening although not identified as such), and these are excluded from the following analysis, as are the 4 women for whom an estimated date of conception was not recorded. The crude probability of conception at 365 days was 45 % (+/− standard deviation 3.5 %) for high non-tryers and 76 % (+/− 3.5 %) for high tryers. The probability of conception adjusted for loss to follow-up, shown in Fig. 2, was about 56 % (+/− 4.0 %) for high non-tryers and 86 % (+/− 2.9 %) for high tryers at one year, respectively. The difference in cumulative probability of conception was significantly different between the groups (*p* < 0.001, log rank test).

### Timeliness of study visit near conception

Of the 165 women enrolled preconception who became pregnant, 161 had dates of conception obtainable from ultrasound; 137 (85 %) of these had a record of completing a preconception visit. (Of the 24 women who were not known to complete a preconception visit, 18 had a conception date after September 2010, i.e., after the change in study data systems, a time frame for which we did not have data regarding study visits available.) As shown in Table 3, only 19 % of these visits took place within 30 days before or after the estimated date of conception. Expanding to 90 days before to 30 days after conception, the proportions are still well less than half: overall 33 %, high tryers 38 %, high non-tryers 22 %. Overall, 39 % occurred more than 90 days prior to conception. Finally, 13 % occurred more than 30 days after conception.

**Table 3:** Time interval from preconception study visit to conception, by eligibility status at the time of consent (*n* = 161)^a^

Time interval relative to conception	High non-tryer	High tryer	All preconception
	%	%	%
More than 90 days before conception	51.9	32.7	39.1
31 to 90 days before conception	9.3	16.8	14.3
30 days before or after conception	13.0	21.5	18.6
More than 30 days after conception	11.1	14.0	13.0
No preconception visit recorded^b^	14.8	15.0	14.9
Total	100	100	100

^a^The table excludes four women with an unknown estimated date of conception

^b^11 % of all preconception women had a conception date after August 2010, a time period for which data on study visits was not available

### Timeliness of first pregnancy study visit

**Fig. 3:**
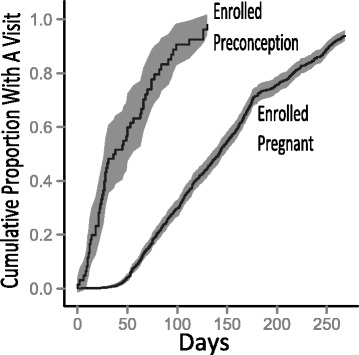
Time from conception to first pregnancy study visit by type of enrollment, adjusted for exiting from study (due to change in eligibility, withdrawal, pregnancy loss, or loss to follow-up). Shading represents 95 % confidence interval

We also examined the time from conception to the first home study visit during pregnancy, in relation to the type of study eligibility at the time of *consent* (Fig. 3). By 10.5 weeks (74 days) after conception, 75 % of women enrolled preconception had completed a home study visit; for women enrolled pregnant, the 75 % threshold was not reached until 28.3 weeks (198 days) after conception. The time to first pregnancy study visit was significantly different between the groups (*p* < 0.001, log rank test). However, among the women enrolled preconception, there was no difference in time to first pregnancy visit between the high tryers and the high non-tryers (data not shown).

## Discussion

The Initial Vanguard Centers of the National Children’s Study enrolled 1399 women, 429 preconception (198 trying for pregnancy, and 231 not trying) and 970 already pregnant, resulting ultimately in 922 births. Preconception women represented 30.6 % of women enrolled, and contributed 14.5 % of births. Among women who gave birth, and who had enrolled preconception trying for pregnancy, 67.3 % were white non-Hispanic, compared to 50.0 % of preconception women giving birth who enrolled not trying for pregnancy. Women enrolled preconception who were trying for pregnancy had higher cumulative probability of pregnancy at one year compared to women not trying (adjusted 86 % versus 56 %). Of 165 women enrolled preconception who became pregnant, 19 % had a study visit within 30 days before or after conception. By 10.5 weeks after conception, 75 % of women enrolled preconception had completed a pregnancy study visit; for women enrolled pregnant, the 75 % threshold was reached at 28.4 weeks.

These results provide important insights for future preconception cohort studies with regard to the proportion of births ultimately enrolled for women enrolled preconception, demographic characteristics of women enrolled trying or not trying for pregnancy, the time to live birth for women enrolled trying or not trying to conceive, and the timeliness of exposure assessments around conception or in early pregnancy. We discuss each of these in turn below.

### Yield and retention

Although approximately one third (31 %) of the women enrolled into the NCS enrolled with preconception status, these women contributed 15 % of the births in the study, with the drop being related to the proportion of preconception women actually conceiving. Because the preconception enrollment part of the protocol was not implemented until nearly a year after the study recruitment began, some of the women who would have been eligible for preconception enrollment early on were instead enrolled later as already pregnant (see Fig. 1, and Table 1, footnote). Therefore the proportion of births from preconception women would most likely have been higher if the preconception enrollment had been implemented from the beginning.

Pregnancy loss was specifically documented for 34 pregnancies (3.0 %), while the incidence of clinically recognized spontaneous abortion in normal pregnancy has been reported at 12–16 % [13, 14]. This strongly suggests that loss was under-ascertained in the follow-up. The data did not allow us to identify what proportion of women who did not proceed to live birth had a pregnancy loss, moved, or were lost to follow-up. However, the combined retention rates were about the same for all pregnant women, regardless of how they were enrolled, mitigating any concern for selection bias resulting from differential retention. We consider this rate of retention to be good, given the high mobility of pregnant women. In prior population-based studies in North America, from 12 to 25 % changed residence during pregnancy [15, 16].

### Demographic characteristics by type of eligibility at consent

Of women giving birth who were enrolled preconception and trying for pregnancy, 67.3 % were white non-Hispanic and 10.2 % Hispanic, compared to preconception women not trying for pregnancy (but heterosexually active and at substantial risk for pregnancy), among whom 50 % were white non-Hispanic and 22.7 % Hispanic (Table 2). Among women who were enrolled pregnant, 61.5 % were white non-Hispanic and 20.0 % Hispanic pregnant women. This is consistent with national U.S. data showing higher proportions of unintended pregnancies among most racial and ethnic minorities [17].

### Time to pregnancy among preconception women

Women who reported they were trying for pregnancy for less than 5 months (high tryers) had cumulative pregnancy rates quite similar to other studies of time to pregnancy in couples without known subfertility, i.e., 86 % at 1 year, adjusting for loss to follow up [18, 19]. Because about half of pregnancies in the United States are reported as unintended [17], the study protocol attempted also to identify those at risk of pregnancy who are not planning pregnancy. The rates of transition to pregnancy among the high non-tryers found in the initial vanguard cohort of the NCS were somewhat lower than in the women trying to become pregnant (56 % at one year versus 86 %, adjusting for loss to follow up). This is not surprising given the criteria used to classify women as high non-tryers. For example, users of traditional calendar rhythm or withdrawal have average first year pregnancy rates of 25 % or less [20]. In older national data, married women who reported intercourse within the last 3 months, no contraceptive use, and no intention to get pregnant had first year pregnancy rates of 57 % or less [21]. Anecdotally, many of the women in the high non-tryer group questioned why they were eligible for preconception enrollment, when they did not consider themselves likely to get pregnant. Yet, over half of this group of women did conceive within a year.

### Timeliness of study visit near conception

One of the reasons for preconception enrollment was to conduct a detailed preconception study assessment in the home, including collecting environmental samples, biological samples, and questionnaires. We were disappointed to find that so few of the the preconception study visits were conducted close to the time of conception: among 165 women enrolled preconception who became pregnant, only 19 % had a study visit within 30 days before or after conception. About a third (33 %) of visits were conducted within 90 days before to 30 days after the date of conception. This proportion was greater for high tryers (38 %) than for high non-tryers (22 %). As noted below, some of the logistics of scheduling likely influenced the timeliness of preconception study visits.

### Timeliness of first pregnancy study visit

Women enrolled preconception were very likely to complete first study visits during pregnancy in a timely manner, much more so than women enrolled during pregnancy. By 10.5 weeks after conception (12.5 weeks gestation age), 75 % of women enrolled preconception had completed a home study visit; for women enrolled pregnant, the 75 % threshold was not reached until 28.3 weeks after conception (30.3 weeks gestational age). This occurred despite the fact that the study was designed to facilitate enrollment as early in pregnancy as possible, and that during the first approximately 3 months of study recruitment, pregnant women had to be with the first 20 weeks of pregnancy to be eligible for the study. The fact that women enrolled preconception had an earlier identification of their pregnancy to study staff facilitated the scheduling of an early study visit.

### Challenges in scheduling study visits

The structure of the study included a number of challenges for scheduling study visits. The staff for enumeration and consent were typically different from the staff for the study visits, and the training for each was role-specific. Initially, different information systems with different field devices were used for enumeration and consent than for study visits. Home study visits were often scheduled on evenings or weekends, to adapt to participant schedules. Calendars needed to be orchestrated for the availability of the personnel and equipment required for each visit.

Pregnancy status was assessed explicitly by questions within the initial screening, and at scheduled follow-up phone calls, as described above. However, study instruments did not include questions addressing current pregnancy status at the time of scheduling for the preconception visit, nor during the preconception visit itself; also a urine pregnancy test was not part of the preconception visit. Despite this potential delay in identifying pregnancy among the women enrolled preconception, they still had their first home study visit earlier than women enrolled during pregnancy.

### Limitations

Our analysis is limited by the transition of the Initial Vanguard Centers to new, decentralized data systems that occurred after September 2010. We did not have access to any information regarding study visits after the transition in data systems. For women exiting the study prior to a live birth, we did not have systematic data on whether the exit was due to a pregnancy loss, a known move, or simply a loss to follow up.

### Strengths

The Initial Vanguard Centers successfully conducted a rich and complex implementation of the protocol for the initial vanguard cohort of the NCS. The sampling frame was well defined. In fact, other Vanguard Centers have reported on more specific comparisons of participants to births within study SSUs based on birth certificates [22]. The rich characterization of both high tryers and high non-tryers provides valuable insight into the characterization and follow up of a population-based sample of women at higher risk of pregnancy.

### Rationale for early exposure assessment

Animal and human studies indicate that exposures in the earliest stages of human development, including the embryonic and early fetal periods, may have a large impact on subsequent development, health and illness throughout the lifespan [23–26], and that the impact of some exposures may be transgenerational by epigenetic mechanisms [27]. Based on this rationale, a number of scientists proposed assessing environmental exposure as early in pregnancy as possible, including preconception and periconceptional periods [28–30]. Because of time-varying components of some exposures, there is high value in conducting such assessment prospectively. For these reasons, the initial vanguard cohort of the NCS was designed to enroll women as early in pregnancy as possible, and also to enroll some women who were not currently pregnant, but at high probability of becoming pregnant in the near future [8]. The importance of timing in exposure assessment will of course vary according to a number of factors involved for each specific exposure, including the likelihood of change of level in a specific exposure over time before and/or after conception, the developmental window of interest for the exposure, and the reliability of measuring the exposure at different time points retrospectively or perhaps prospectively in relation to the time window of interest.

### Competing priorities

In recent years, there has been a flourishing of new birth cohorts, as well as new data from long-established birth cohorts, seeking to address questions regarding early life course exposures and the developmental origins of childhood and adult disease [31, 32]. Before discontinuation of the NCS, there was extensive discussion of the best way to realize its potential, including recommendations to abandon preconception recruitment [10, 33], maintain preconception recruitment to the extent possible [34], maintain probability-based household sampling [35], move to provider-based sampling [22, 36], focus on explicit hypotheses for important diseases of low incidence [37], and so on. All such suggestions involve choices and trade-offs between competing priorities and the impact of different types of recruitment for any birth cohort study [38, 39]. Our analysis provides data informing some of these competing priorities in reference to assessment of peri-conception and early pregnancy exposures.

To the extent that the goal for a pregnancy and birth cohort study may be robust preconception exposure assessment in an efficient manner, a logical focus would be on recruiting women or couples trying for pregnancy, while still maintaining a population-based sampling frame. This could be done while considering more cost-effective recruiting mechanisms than door-to-door recruitment, including mail or internet [30, 40], more streamlined preconception exposure assessment, as well as innovative low-cost approaches to identifying the preconception period [41, 42]. There is a trade-off that women trying to conceive are demographically significantly different than other women who conceive without “trying”, which may limit external generalizability.

Our results also suggest that preconception recruitment can help achieve the goal of exposure assessment early in pregnancy, more so than recruitment during pregnancy. With regard to the timing of the first study visit in pregnancy, the experience of high tryers and high non-tryers was identical, suggesting that the decision of whether to include high non-tryers in a cohort would have to weigh representativeness against efficiency and resources.

Provider-based sampling and recruitment, while presenting a number of valuable efficiencies and retaining overall representativeness [36, 43], may present challenges for early exposure assessment. Women in the United States do not obtain prenatal care as early as in other countries that have conducted pregnancy cohorts [44, 45]. Minority women and women who are less compliant with medical procedures present substantially later for prenatal care [45–47]. In the United States, preconception care from providers is recommended to be integrated into primary care for both women and men, but there are no measures to identify the extent to which this is happening, or to which provider based sampling could be effectively used for preconception recruitment [48].

Finally, pregnancy to birth cohort studies should carefully track pregnancy outcomes other than live birth, not only to understand the reasons for attrition, but also because a complete analysis and understanding of the impact of environmental exposures on early human development requires the inclusion of human developmental outcomes other than live birth [49, 50].

## Conclusions

The Initial Vanguard Centers of the U.S. National Children’s Study successfully recruited and enrolled 1399 women in pregnancy (69 %) or preconception (31 %), while exploring many of the competing priorities and difficult decisions that need to be addressed in future research. These data confirm that there are important demographic differences in births from women who try to conceive and women who do not try to conceive; and that it is possible to enroll and follow women preconception who are at substantial risk of pregnancy, despite the fact that they are not trying to conceive. While time to pregnancy is shorter for women actively trying for pregnancy, approximately half of women who are not trying but are at substantial risk for pregnancy conceive within a year. In the context of the protocol for the initial vanguard cohort of the NCS, most women enrolled preconception did not have exposure assessment within 30 days of conception, but they did have exposure assessment much earlier during pregnancy than women who enrolled during pregnancy.

## References

[1] Hirschfeld S, Kramer B, Guttmacher A (2010). Current status of the national Children’s study. Epidemiology.

[2] Branum AM, Collman GW, Correa A, Keim SA, Kessel W, Kimmel CA (2003). The National Children’s Study of environmental effects on child health and development. Environ Health Perspect.

[3] Montaquila JM, Brick JM, Curtin LR (2010). Statistical and practical issues in the design of a national probability sample of births for the Vanguard Study of the National Children’s Study. Stat Med.

[4] NIH cancels massive U.S. children’s study [http://news.sciencemag.org/funding/2014/12/nih-cancels-massive-u-s-children-s-study]

[5] Mortensen ME, Hirschfeld S (2012). The National Children’s Study: an opportunity for medical toxicology. J Med Toxicol.

[6] Guttmacher AE, Hirschfeld S, Collins FS (2013). The National Children’s Study--a proposed plan. N Engl J Med.

[7] Landrigan PJ, Trasande L, Thorpe LE, Gwynn C, Lioy PJ, D’Alton ME (2006). The National Children’s Study: a 21-year prospective study of 100,000 American children. Pediatrics.

[8] Selevan SG, Stanford JB (2006). Workshop recommendations for the preconception cohort of the National Children’s Study. Paediatr Perinat Epidemiol.

[9] Buck GM, Lynch CD, Stanford JB, Sweeney AM, Schieve LA, Rockett JC (2004). Prospective pregnancy study designs for assessing reproductive and developmental toxicants. Environ Health Perspect.

[10] Savitz DA, Ness RB (2010). Saving the National Children’s Study. Epidemiology.

[11] Baker D, Park C, Sweeney C, McCormack L, Durkin M, Brenner R (2014). Recruitment of women in the National Children’s Study initial vanguard study. Am J Epidemiol.

[12] National Research Council, Institute of Medicine. The national children’s study research plan: a review. Washington (DC): National Academies Press; 2008.

[13] Wilcox AJ, Weinberg CR, O’Connor JF, Baird DD, Schlatterer JP, Canfield RE (1988). Incidence of early loss of pregnancy. N Engl J Med.

[14] Mills JL, Simpson JL, Driscoll SG, Jovanovic-Peterson L, Van Allen M, Aarons JH (1988). Incidence of spontaneous abortion among normal women and insulin-dependent diabetic women whose pregnancies were identified within 21 days of conception. N Engl J Med.

[15] Fell DB, Dodds L, King WD (2004). Residential mobility during pregnancy. Paediatr Perinat Epidemiol.

[16] Shaw GM, Malcoe LH (1992). Residential mobility during pregnancy for mothers of infants with or without congenital cardiac anomalies: a reprint. Arch Environ Health.

[17] Finer LB, Zolna MR. Shifts in intended and unintended pregnancies in the United States, 2001–2008. Am J Public Health. 2014;104(Suppl 1):S43-8. 10.2105/AJPH.2013.301416 PMC4011100 24354819

[18] Wang X, Chen C, Wang L, Chen D, Guang W, French J (2003). Conception, early pregnancy loss, and time to clinical pregnancy: a population-based prospective study. Fertil Steril.

[19] Dunson DB, Baird DD, Colombo B (2004). Increased infertility with age in men and women. Obstet Gynecol.

[20] Trussell J (2004). Contraceptive failure in the United States. Contraception.

[21] Grady WR, Hayward MD, Yagi J (1986). Contraceptive failure in the United States: estimates from the 1982 national survey of family growth. Fam Plann Perspect.

[22] Kerver JM, Elliott MR, Norman GS, Sokol RJ, Keating DP, Copeland GE (2013). Pregnancy recruitment for population research: the National Children’s Study vanguard experience in Wayne County, Michigan. Paediatr Perinat Epidemiol.

[23] Kwong WY, Wild AE, Roberts P, Willis AC, Fleming TP (2000). Maternal undernutrition during the preimplantation period of rat development causes blastocyst abnormalities and programming of postnatal hypertension. Development.

[24] Hernandez CE, Matthews LR, Oliver MH, Bloomfield FH, Harding JE (2010). Effects of sex, litter size and periconceptional ewe nutrition on offspring behavioural and physiological response to isolation. Physiol Behav.

[25] Hochberg Z, Feil R, Constancia M, Fraga M, Junien C, Carel JC (2011). Child health, developmental plasticity, and epigenetic programming. Endocr Rev.

[26] Gillman MW, Barker D, Bier D, Cagampang F, Challis J, Fall C (2007). Meeting report on the 3rd international congress on Developmental Origins of Health and Disease (DOHaD). Pediatr Res.

[27] Ziv-Gal A, Wang W, Zhou C, Flaws JA (2015). The effects of in utero bisphenol A exposure on reproductive capacity in several generations of mice. Toxicol Appl Pharmacol.

[28] Selevan SG, Kimmel CA, Mendola P (2000). Identifying critical windows of exposure for children’s health. Environ Health Perspect.

[29] Chapin RE, Buck GM (2004). Our once-in-a-lifetime opportunity. Environ Health Perspect.

[30] Buck Louis GM, Schisterman EF, Sweeney AM, Wilcosky TC, Gore-Langton RE, Lynch CD (2011). Designing prospective cohort studies for assessing reproductive and developmental toxicity during sensitive windows of human reproduction and development--the LIFE study. Paediatr Perinat Epidemiol.

[31] Vrijheid M, Casas M, Bergstrom A, Carmichael A, Cordier S, Eggesbo M (2012). European birth cohorts for environmental health research. Environ Health Perspect.

[32] van Gelder MM, Bretveld RW, Roukema J, Steenhoek M, van Drongelen J, Spaanderman ME (2013). Rationale and design of the PRegnancy and Infant DEvelopment (PRIDE) study. Paediatr Perinat Epidemiol.

[33] Landrigan PJ, Baker DB (2015). The National Children’s Study--end or new beginning?. N Engl J Med.

[34] Olsen J (2013). Random sampling - is it worth it?. Paediatr Perinat Epidemiol.

[35] Michael RT, O’Muircheartaigh CA (2010). US National Children’s Study. Epidemiology.

[36] Belanger K, Buka S, Cherry DC, Dudley DJ, Elliott MR, Hale DE (2013). Implementing provider-based sampling for the National Children’s Study: opportunities and challenges. Paediatr Perinat Epidemiol.

[37] Paneth N (2013). Restoring science to the National Children’s Study. JAMA.

[38] Savitz DA (2013). Sample selection for the National Children’s Study: form must follow function. Paediatr Perinat Epidemiol.

[39] Maghera A, Kahlke P, Lau A, Zeng Y, Hoskins C, Corbett T (2014). You are how you recruit: a cohort and randomized controlled trial of recruitment strategies. BMC Med Res Methodol.

[40] Huybrechts KF, Mikkelsen EM, Christensen T, Riis AH, Hatch EE, Wise LA (2010). A successful implementation of e-epidemiology: the Danish pregnancy planning study ‘Snart-Gravid’. Eur J Epidemiol.

[41] Zinaman MJ (2006). Using cervical mucus and other easily observed biomarkers to identify ovulation in prospective pregnancy trials. Paediatr Perinat Epidemiol.

[42] Porucznik CA, Cox KJ, Schliep KC, Stanford JB (2014). Pilot test and validation of the Peak Day method of prospective determination of ovulation against a handheld urine hormone monitor. BMC Womens Health.

[43] Hirschfeld S (2013). DRAFT main study protocol outline of the National Children’s Study, version 4.0. United States, National Institutes of Health.

[44] Olsen J, Melbye M, Olsen SF, Sorensen TI, Aaby P, Andersen AM (2001). The Danish National Birth Cohort--its background, structure and aim. Scand J Public Health.

[45] Martin JA, Hamilton BE, Ventura SJ, Osterman MJ, Kirmeyer S, Mathews TJ (2011). Births: final data for 2009. Natl Vital Stat Rep.

[46] Alexander GR, Kogan MD, Nabukera S (2002). Racial differences in prenatal care use in the United States: are disparities decreasing?. Am J Public Health.

[47] Sacks D (2003). Fasting plasma glucose test at the first prenatal visit as a screen for gestational diabetes. Obstet Gynecol.

[48] Johnson K, Posner SF, Biermann J, Cordero JF, Atrash HK, Parker CS (2006). Recommendations to improve preconception health and health care--United States. A report of the CDC/ATSDR preconception care work group and the select panel on preconception care. MMWR Recomm Rep.

[49] Joseph KS, Demissie K, Platt RW, Ananth CV, McCarthy BJ, Kramer MS (2004). A parsimonious explanation for intersecting perinatal mortality curves: understanding the effects of race and of maternal smoking. BMC Pregnancy Childbirth.

[50] Paneth N (2008). Invited commentary: the hidden population in perinatal epidemiology. Am J Epidemiol.

